# Description of the Serological Response After Treatment of Chronic Imported Schistosomiasis

**DOI:** 10.3390/tropicalmed10010022

**Published:** 2025-01-14

**Authors:** Marta González-Sanz, Irene Martín-Rubio, Oihane Martín, Alfonso Muriel, Sagrario de la Fuente-Hernanz, Clara Crespillo-Andújar, Sandra Chamorro-Tojeiro, Begoña Monge-Maíllo, Francesca F. Norman, José A. Pérez-Molina

**Affiliations:** 1 National Reference Centre for Imported Tropical Diseases, Infectious Diseases Department, Hospital Universitario Ramón y Cajal, IRYCIS, 28034 Madrid, Spain; irenemartinrubio@yahoo.es (I.M.-R.);; 2 Departamento de Medicina y Especialidades Médicas, Programa de Ciencias de la Salud, Universidad de Alcalá, 28801 Alcalá de Henares, Spain; 3 CIBERINFEC, Instituto de Salud Carlos III, 28029 Madrid, Spain; 4 National Reference Centre for Imported Tropical Diseases, Microbiology Department, Hospital Universitario Ramón y Cajal, IRYCIS, 28034 Madrid, Spain; 5 Clinical Biostatistic Unit, Hospital Universitario Ramón y Cajal, IRYCIS, 28034 Madrid, Spain; 6 CIBERESP, Instituto de Salud Carlos III, 28029 Madrid, Spain

**Keywords:** schistosomiasis, migrant health, neglected tropical diseases, diagnosis, serology, travelers

## Abstract

Background: Chronic schistosomiasis can lead to significant morbidity. Serology is highly sensitive; however, its role in assessing treatment response is controversial. This study aimed to analyze serological values following treatment of chronic imported schistosomiasis. Methods: A retrospective observational study was performed including patients treated for chronic imported schistosomiasis from 2018 to 2022 who had at least one serological result at baseline and during follow-up. Demographic, clinical, and laboratory data were evaluated. Generalized estimating equation (GEE) models and Kaplan–Meier curves were used to analyze the evolution of serological values. Results: Of the 83 patients included, 72 (86.7%) were male, and the median age was 26 years (IQR 22–83). Most patients, 76 (91.6%), were migrants from sub-Saharan Africa. While 24 cases (28.9%) presented with urinary symptoms, the majority (59; 71.1%) were asymptomatic. *Schistosoma haematobium* eggs were observed in five cases (6.2%). Eosinophilia was present in 34 participants (40.9%). All patients had an initial positive *Schistosoma* ELISA serology, median ODI 2.3 (IQR 1.5–4.4); the indirect hemagglutination (IHA) test was positive/indeterminate in 34 cases (43.1%). Following treatment with praziquantel, serology values significantly decreased: −0.04 (IC95% −0.073, −0.0021) and −5.73 (IC95% −9.92, −1.53) units per month for ELISA and IHA, respectively. A quarter of patients (25%) had negative ELISA results 63 weeks after treatment. All symptomatic cases were clinically cured. Conclusions: Serial serological determinations could be helpful for monitoring chronic schistosomiasis in non-endemic regions. The ideal timing for these follow-up tests is yet to be determined. Further research is needed to determine the factors that influence a negative result during follow-up.

## 1. Introduction

Schistosomiasis is a neglected tropical disease with a great impact on health and social well-being, mainly affecting populations of low socioeconomic status. The World Health Organization (WHO) estimated there are more than 250 million people requiring preventive treatment for schistosomiasis, over 90% of them living in sub-Saharan Africa. The disease is endemic in 78 countries [1,2]. Diagnosis of this parasitosis among migrants and travelers is common, making it a public health and clinical challenge [3,4,5]. In addition to the potential difficulties in diagnosing and managing imported schistosomiasis, there is a real risk of reintroducing this disease in Europe [6,7,8].

Travel and migration have contributed to the spread of schistosomiasis to non-endemic areas. The seroprevalence of schistosomiasis in migrants from endemic areas is estimated to be 18.4%, rising to 24.1% in migrants from sub-Saharan Africa [4]. The most prevalent clinical form in this population is chronic schistosomiasis, resulting from repeated exposure to the parasite. The deposition of eggs in the urinary or intestinal tracts leads to a chronic inflammation responsible for morbidity and complications [2,9]. Schistosomiasis accounts for up to 3.8% of all travel-related diseases diagnosed in returning travelers [5]. *Schistosoma* spp. infections in travelers and expatriates account for 49% of all imported cases of schistosomiasis, most of whom have acquired the parasite in sub-Saharan Africa [10]. The majority of infections in travelers are symptomatic (57.5%), unlike those occurring in migrants, and in these, 27% present symptoms within three months of return [10].

Chronic schistosomiasis is a diagnostic challenge as it is often asymptomatic or presents with eosinophilia only [10,11,12]. If untreated, complications include bladder wall focal or diffuse thickening, obstructive uropathy with ureter dilatation and hydronephrosis, squamous cell carcinoma of the bladder and female genital schistosomiasis in the case of *S. haematobium*, and liver fibrosis with portal hypertension and esophageal varices, colorectal ulcerations, and polyps in the case of *S. mansoni* [2,3,9,13,14,15,16].

Active schistosomiasis is diagnosed following detection of viable eggs in feces, urine, or tissue samples. These techniques show very low sensitivity, though high specificity, which precludes their use as a test of cure, especially with low parasite burdens [17]. Molecular tests to identify DNA in clinical samples have a higher sensitivity but still need standardization, may suffer from sampling limitations, take time to become negative after treatment, are expensive, and may not be affordable for some clinical laboratories [17,18,19,20]. Schistosoma antigen detection is a highly sensitive assay (both cathodic (CCA) and anodic circulating antigens (CAA)), which can be detected in serum, plasma, and urine [21,22,23,24]. A commercially available cathodic antigen (CCA)-based rapid test for detecting *S. mansoni* antigens in urine that is more sensitive than the Kato–Katz technique is also available and is recommended by WHO for detecting and mapping this parasite in endemic regions [25,26]. CAA especially allows quantification after treatment [23,27]. However, the main disadvantages of these tests are that CCA is mainly useful for *S. mansoni* only and CAA is not commercially available. This makes serology the most widely used diagnostic method. It is very sensitive, especially if two techniques are used simultaneously, specific, widely available, and inexpensive. Main disadvantages are that serology does not differentiate between species or between active infection, past infection, or reinfection, as the disappearance of antibodies after treatment may take years or not occur at all [17,24,28,29].

The aim of this study was to describe the characteristics of patients diagnosed with chronic imported schistosomiasis at a reference center in Spain and analyze their serological and eosinophil response following treatment with praziquantel.

## 2. Materials and Methods

### 2.1. Study Design and Study Population

An observational retrospective study was carried out at the National Reference Centre for Imported Tropical Diseases, Ramón y Cajal University Hospital, Madrid, Spain, from January 2018 to January 2022. Patients with a diagnosis of chronic imported schistosomiasis were included. Cases of acute schistosomiasis were excluded. Patients were classified into three groups: travelers (including migrant travelers visiting friends and relatives (VFR)), expatriates, and migrants. All patients had an initial positive serological test, had received treatment with praziquantel, and had at least one serological determination at follow-up.

Demographic (age, sex, country of origin, and type of patient), clinical (presence or absence of symptoms), laboratory (eosinophilia, direct parasitological tests, *Schistosoma* spp. ELISA and indirect hemagglutination (IHA) test, HIV and *Strongyloides* serology), and treatment data were registered. Information was collected at baseline and at each subsequent follow-up visit, regardless of the number of visits.

The primary endpoint was to describe the variation in antibody values against *Schistosoma* spp. after anti-parasitic treatment. Further subanalysis evaluated the association between the change in antibody values and sociodemographic variables such as age, sex, type of patient (traveler, VFR, expatriate, or migrant), and days until diagnosis after travel (for travelers and expatriates) or arrival to Spain (for migrants).

### 2.2. Definitions and Microbiological Techniques

Cases of schistosomiasis were classified as follows (certainty of diagnosis):

1. Confirmed schistosomiasis, diagnosed by direct microscopy with visualization of eggs in urine, feces, or tissue samples.
2. Probable schistosomiasis; cases with clinical symptoms or signs of disease (eosinophilia, hematuria, or visceral involvement) with positive serology.
3. Possible schistosomiasis, positive serology in the absence of symptoms or signs of disease.

Eosinophilia was defined as an eosinophil cell count ≥ 450 cells/mm^3^ or a percentage ≥ 5%. Microscopic examination for ova detection was carried out after concentration of three stool samples collected on alternate days (Mini PARASEP^®^ Alcorfix APACOR, Wokingham, UK) and filtration of at least 10 mL of urine using a 10 µm pore membrane (Isopore polycarbonate membrane, Merck Millipore, Carrigtwohill, Ireland).

A combination of two commercial tests was used for serological diagnosis: IgG antibodies against *Schistosoma* spp. were determined by ELISA (*Schistosoma mansoni* IgG NovaLisa^®^ NovaTec Immunodiagnostica, GMBH, Dietzenbach, Germany) and an indirect hemagglutination assay (SCHISTOSOMIASIS FUMOUZE^®^, ELITech Group, Signes, France). An optical density index (ODI) above 1.1 was considered positive for the ELISA test, and a titer ≥ 1/160 was positive for IHA (indeterminate result: 1/80).

Serological response to treatment was defined as a negative serology result during follow-up (regardless of when patients reached this status) or a decrease of at least 50% in the ODI for ELISA or a four-fold decrease in IHA test titers.

### 2.3. Ethical Issues

All cases were identified from the existing database at the National Reference Centre for Tropical Diseases. Data were incorporated into the database after patients signed an informed consent form. This study was granted approval by the Hospital Universitario Ramón y Cajal Institutional Review Board (Minute 433; 17 May 2022).

### 2.4. Statistical Analysis

Categorical data were presented as absolute numbers and proportions, and continuous variables were expressed as medians and interquartile ranges (IQR). The χ^2^ test, or Fisher exact test, when appropriate, was used to compare the distribution of categorical variables, and the t-Student test for continuous variables. Time to serological seroreversion and time to a decrease of 50% in serological values were collected for all study participants, and the Kaplan–Meier survival curve was generated to display the probability of such events over the study period. The log-rank test was used to compare the equality of survival functions. Generalized Estimating Equations (GEE) were used to analyze serological values over time, which accounts for the within-subject correlation over time. The models were performed only to analyze changes in ELISA values because the number of determinations and events with the indirect hemagglutination assay was much smaller. The GEE method estimates the population-averaged effect of the independent variables on the outcome and allows for the inclusion of time-varying covariates. An estimative approach was employed to model the data in the study. Estimated coefficients and their corresponding *p*-values were reported. A two-tailed 5% level of significance was set. Statistical analysis was carried out using Stata ver. 17 (StataCorp. 2021. Stata Statistical Software: Release 17. College Station, TX, USA: StataCorp LLC.).

## 3. Results

A total of 348 patients with a diagnosis of chronic imported schistosomiasis were screened, and 83 (23.9%) (Table 1), whose characteristics did not differ significantly from excluded cases (Supplemental Table S1), were included in the study. The main reasons for exclusion (*n* = 265) were lack of serological test at diagnosis (1; 0.4%), indeterminate ELISA result at diagnosis (15; 5.7%), lack of serology test performed at follow-up (110; 41.5%), and loss to follow-up after treatment (139; 52.5%).

Of the 83 included cases, the majority were male (72; 86.7%) with a median age of 26 years (IQR 22–83) and a median follow-up time of 29.7 weeks (IQR: 16.7 to 45.0 weeks). Most were migrants of Sub-Saharan origin (76; 91.6%), Mali being the most represented country of exposure (27; 32.5%).

Fifty-nine patients (71.1%) were asymptomatic at diagnosis, and the rest, 24 (28.9%), had urinary symptoms. Eosinophilia was present in 34 participants (41%); 32 (38.6%) had relative eosinophilia, whilst only around a quarter of patients (21/82, 25.3%) presented with absolute eosinophilia. HIV co-infection was diagnosed in one patient (1/79, 1.3%), whilst *Strongyloides* co-infection was present in a quarter of patients (20/80, 25%).

Direct parasitological diagnosis was positive in only 5 out of 81 cases tested (6.2%), with *Schistosoma haematobium* eggs being demonstrated in urine. ELISA serology for *Schistosoma* spp. was positive for all patients with a median optical density index (ODI) of 2.3 (IQR 1.5–4.4). Indirect hemagglutination (IHA) was positive/indeterminate in 34 out of 79 cases in which it was performed (43%) with median titers of 1/40 (IQR 1/40–1/160).

**Figure 1:**
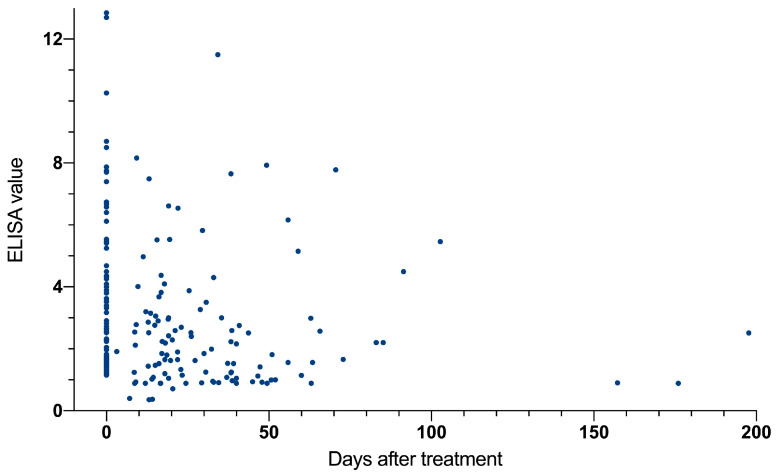
Evolution of ELISA serological values following treatment (in days).

All included patients received treatment with praziquantel at doses between 40 and 60 mg/kg/day (for two cases the exact dose was not recorded). Following treatment, serology values significantly decreased across the study period: −0.04 (IC95% −0.073, −0.0021) and −5.73 (IC95% −9.92, −1.53) units per month for ELISA and IHA, respectively (Figure 1). Absolute and relative numbers of eosinophils also decrease significantly per month: −8.23 cells/μL (IC95% −13.4, −3.01) and −0.16% (IC95% −0.23, −0.90), respectively.

**Figure 2:**
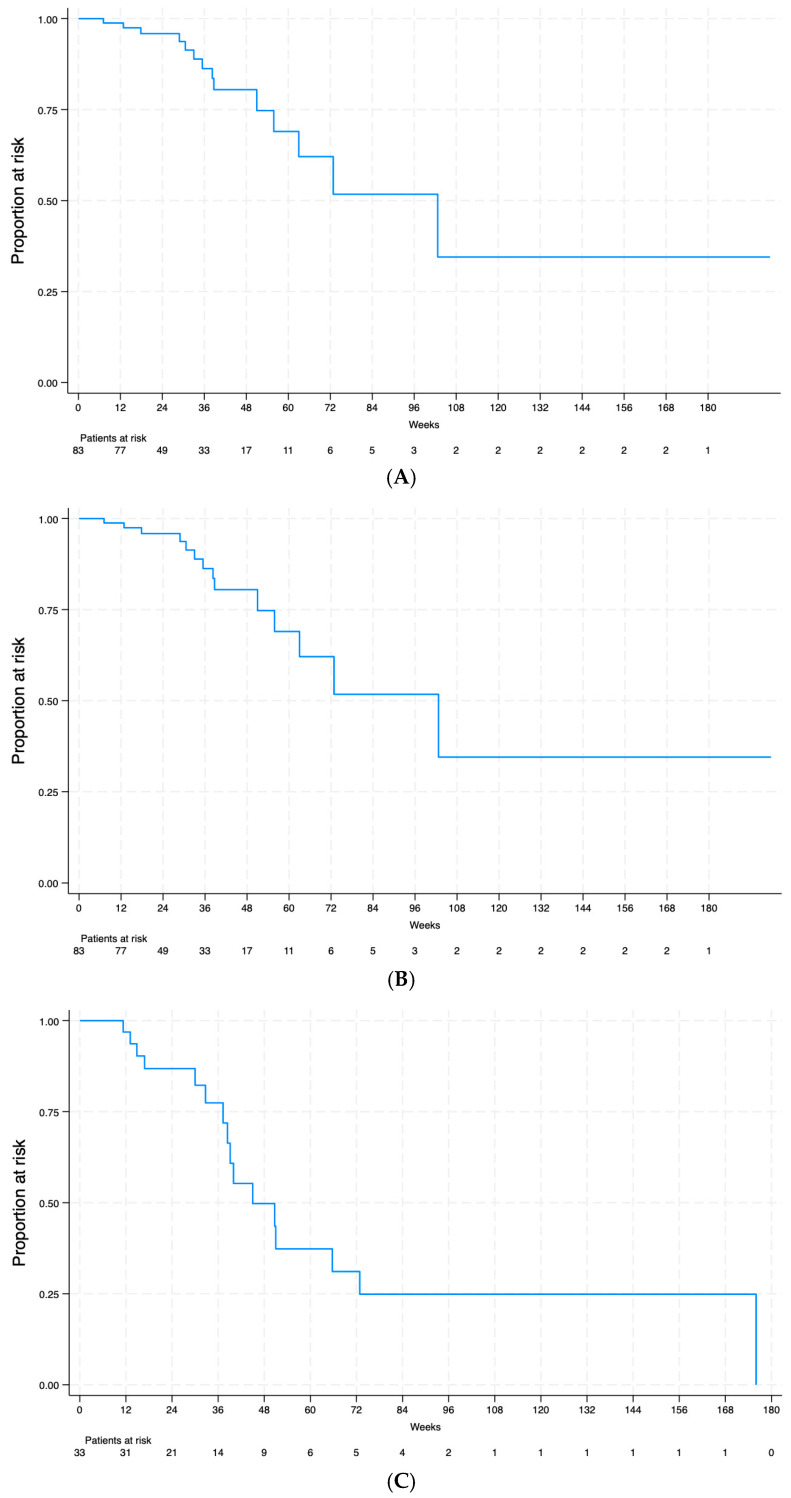
Kaplan–Meier survival curves showing the decrease in serological values following treatment of chronic imported schistosomiasis. (**A**) Time (in weeks) to 50% decrease in ELISA values. (**B**) Time (in weeks) to negative ELISA results. (**C**) Time (in weeks) to negative hemagglutination.

A quarter of patients had a 50% decrease in ELISA values 51 weeks after treatment and half after 102 weeks (Figure 2A). Similarly, a quarter of cases had negative ELISA results 63 weeks after treatment and half after 176 weeks (Figure 2B). However, median estimation was severely affected by the low number of patients at risk. Neither the time to a 50% decrease in ELISA values nor the time to negative serological results was significantly different in terms of diagnostic certainty (*p* = 0.42 and *p* = 0.49, respectively). Regarding the decrease in IHA titers, median time to seroreversion was 45 weeks, while 25% of patients had negative results at 37 weeks of follow-up (Figure 2C). Changes in the IHA titers were unrelated to diagnostic certainty (*p* = 0.13 and *p* = 0.26 for time to seroreversion or a fourfold decrease, respectively).

To estimate the association between potentially related variables with the decrease in antibody values measured by ELISA or eosinophil count, two GEE maximum models with the following independent variables: sex, age, type of patient (traveler, VFR, expatriate, or migrant), and certainty of diagnosis, were developed. After adjusting for potential confounding, no significant relationships were detected between these variables and the decrease in antibody values or eosinophil count.

## 4. Discussion

This study demonstrated a decrease in antibody values against *Schistosoma* spp. and eosinophil counts following treatment of chronic imported schistosomiasis. Changes in these two parameters were unrelated to sex, age, type of patient, or the certainty of diagnosis. While eosinophil numbers may be affected by concomitant parasitoses and their treatment, serological results appear to reflect response to praziquantel treatment more reliably. Therefore, serology could be a helpful tool in the patient’s follow-up and the evaluation of treatment efficacy.

The current study included a large number of patients with prolonged serological follow-up after praziquantel treatment. A robust method for analyzing antibody values and eosinophil counts was used to measure the magnitude of variations, therefore permitting the identification of subtle but significant differences. The population, with a high proportion of migrants, is representative of imported cases of chronic schistosomiasis in Europe, making the results more easily generalizable. As the study was conducted in a non-endemic area, the absence of reinfection, facilitates the interpretation of serologic test results. Lastly, although there is no consensus on the different levels of certainty for diagnosing chronic schistosomiasis, easily interpretable definitions were used, allowing comparison of the study data with other studies.

The limitations of this study include the retrospective design, which made it more prone to bias. Although 25% of participants were followed for 45 months or more, there were many losses after seven months of follow-up due to the geographical mobility of migrants. The analysis of eosinophilia is challenging to interpret, as the presence and treatment of another concomitant parasitosis may have affected the observed variations. Although no relationship was found between sex, age, type of patient, or certainty of diagnosis, it is possible that the number of seroreversions or subjects analyzed was insufficient to detect small differences.

Except for the retrospective study by Whitty et al. [30], which included 276 patients with chronic schistosomiasis and serological follow-up after treatment with praziquantel, more recent studies in non-endemic areas have included a lower number of subjects, ranging from 24 to 58 participants [23,28,31]. In these studies, most of the study population were migrants with a follow-up time of 12 months, except for Yong et al. [28], in whose work 44.8% were travelers, and serological follow-up was up to 30 months. A significant decrease in serological values was observed in all these studies except in the study by Tamarozzi et al. [23]. The serological response, or the proportion of seroreversion, ranged from 24% to 68%, becoming higher as follow-up increased [28,30,31]. Nevertheless, such serological responses were not homogeneous. Some subjects did not show changes after treatment, with some showing transient elevations during the first 3–5 months. In this study, the 83 patients included, and the prolonged follow-up may have served to identify variations not detected in other studies. These variations in serological response may result from multiple factors such as the type of patient (travelers vs. migrants), age, time since infection, the serological technique, or the treatment regimen used (single vs. multiple doses). In this way, Yong et al. found significantly better serological responses in travelers than migrants [28]. In the current study, IHA post-treatment results became negative quicker than with ELISA, but less than half of the patients had a positive IHA at diagnosis. The potential role of some prognostic variables in the serological response was analyzed, but significant differences in the response rate according to sex, age, type of patient, or the certainty of diagnosis were not detected. The optimal time to repeat serology following treatment remains unclear, although this study suggests that for most patients, more than one year might be appropriate, which poses a great challenge for migrant populations with high mobility and often a high rate of loss to follow-up.

Schistosomiasis represents a health problem in Europe that deserves attention because of the morbidity and severe complications it causes in chronically infected subjects and because of the possibility of reintroduction in the European continent since, in most cases, it is asymptomatic and can go unnoticed [6,7,8,32]. In fact, autochthonous transmission in Europe has already been documented. The first evidence of local schistosomiasis was identified in Portugal between 1920 and 1967 [33]. In 2013 an outbreak was reported involving more than 100 people in Corsica Island (Cavu River), which is still ongoing [6] and has spread to other rivers in Corsica (Solenzana River) [7,34]. In Spain, an outbreak of autochthonous schistosomiasis (*S. haematobium*) which started in 2003, has recently been reported [8]. The presence of the intermediate host *Bulinus* spp. snails has also been demonstrated in Greece, Cyprus, the south of France, and Sardinia, as well as in other Spanish areas besides Almería [6,33,35]. Climate change might be contributing to a further expansion of the distribution area of the snails [36,37]. Therefore, high awareness of the disease, early screening, and good monitoring of treatment efficacy are of high importance.

Techniques such as PCR or the determination of circulating CAA may be more sensitive than serology in determining response to treatment. However, problems that currently preclude their use in routine clinical practice include cost, the need for standardization, slow decrease in antibody values after treatment, lack of commercial availability, or unsuitability for follow-up [17,27,31].

## 5. Conclusions

Serology is a widely used, standardized, and inexpensive technique and will continue to be a valuable tool for diagnosis and follow-up of chronic schistosomiasis. Studies with larger numbers of patients and longer follow-up are needed to identify groups of patients who may benefit most from using serology as a test of cure. A consensus on diagnostic and therapeutic response criteria for chronic schistosomiasis outside endemic areas should be prioritized.

## Figures and Tables

**Table 1:** Baseline characteristics, clinical presentation, diagnosis, and management of patients with chronic imported schistosomiasis.

	**N = 83**
Age (years); median (IQR)	26 (22–33)
Male sex; *n* (%)	72 (86.7)
Country of acquisition; *n* (%) Mali Ivory Coast Republic of Guinea (Guinea-Conakry) Senegal Sudan Cameroon Gambia Ghana Democratic Republic of Congo Equatorial Guinea Morocco Nigeria	27 (32.5) 9 (10.8) 8 (9.6) 8 (9.6) 4 (4.8) 3 (3.6) 3 (3.6) 2 (2.4) 1 (1.2) 2 (2.4) 2 (2.4) 2 (2.4)
Type of patient; *n* (%) Migrant Traveler VFR-migrant VFR-traveler	76 (91.6) 5 (6) 1 (1.2) 1 (1.2)
Symptoms; *n* (%)	24 (28.9)
Asymptomatic Urinary uncomplicated Urinary complicated Intestinal	59 (71.1) 21 (25.3) 3 (3.6) 0 (0)
Parasitological diagnosis *; *n* (%) Positive urine Positive feces	5 (6.2) 0 (0)
Serological diagnosis ELISA; ODI median (IQR) Positive (≥1.1) IHA ^†^, median (IQR) Positive (≥1/160); *n* (%) Indeterminate (1/80); *n* (%) Negative (≤1/40); *n* (%)	2.3 (1.5–4.4) 83 (100) 1/40 (1/40–1/160) 24 (30.4) 10 (12.7) 45 (57)
Eosinophilia; *n* (%)	34 (40.9)
Absolut (≥450 cel/mm^3^) ^‡^ Relative (≥5%)	21 (25.6) 32 (38.6)
Time to diagnosis (days); median (IQR)	164 (80–260)
Co-infection; *n* (%) HIV ^†^ Strongyloides ^§^	1 (1.3) 20 (25)
Type of diagnosis Confirmed (parasitological diagnosis) Probable (signs or symptoms and positive serology) Possible (asymptomatic and positive serology)	5 (6) 44 (53) 34 (41)
Treatment (mg/kg/24–48 h) ^§^; *n* (%) 60 mg 40 mg Dose not recorded	69 (86.3) 11 (13.8) 2 (2.4)

* % calculated for an available N of 81. ^†^ % calculated for an available N of 79. ^‡^ % calculated for an available N of 82. ^§^ % calculated for an available N of 80. Abbreviations: VFR—visiting friends and relatives; ODI—optical density index; IHA—indirect hemagglutination test.

## Data Availability

The raw data supporting the conclusions of this article will be made available by the authors on request.

## Supplementary Materials

The following supporting information can be downloaded at: https://www.mdpi.com/article/10.3390/tropicalmed10010022/s1, Table S1: Baseline characteristics os included and excluded patients with imported chronic schistosomiasis.
