# Supplementary material for: Description of the Serological Response After Treatment of Chronic Imported Schistosomiasis
Source: Trop Med Infect Dis. 2025 Jan 14;10(1):22. doi: 10.3390/tropicalmed10010022 (PMC11768958; doi:10.3390/tropicalmed10010022)
Supplement: Supplementary file 1 [file tropicalmed-10-00022-s001.zip › tropicalmed-3326941-supplementary.pdf]

Supplemental Table S1. Baseline characteristics os included and excluded patients with imported chronic schistosomiasis

Baseline characteristics	EXCLUDED (N = 265)	INCLUDED (N = 83)	p
Age (years), median (IQR)	26 (22 – 34)	26 (22 – 33)	0.09
Male sex; n (%)	247 (93.2)	72 (86.7)	0.06
Country of acquisition; n (%)			0.49
Mali	70 (26.4)	27 (32.5)	
Ivory Coast	29 (10.9)	9 (10.8)	
Republic of Guinea (Guinea-	47 (17.7)	8 (9.6)	
Conakry)	34 (12.8)	8 (9.6)	
Senegal	6 (2.3)	4 (4.8)	
Sudan	13 (4.9)	3 (3.6)	
Cameroon	10 (3.8)	3 (3.6)	
Gambia	3 (1.1)	2 (2.4)	
Ghana	4 (1.5)	1 (1.2)	
Democratic Republic of Congo	4 (1.5)	2 (2.4)	
Equatorial Guinea	8 (3)	2 (2.4)	
Morroco	2 (0.8)	2 (2.4)	
Nigeria	4 (1.5)	1 (1.2)	
Burkina Faso			
Type of patient; n (%)			0.54
Migrant	244 (92.1)	76 (91.6)	
Traveler	9 (3.4)	5 (6.0)	
VFR-migrant	9 (3.4)	1 (1.2)	
VFR-traveler	3 (1.1)	1 (1.2)	

Abbreviations: VFR- visiting friends and relatives.
